# Modelos Geométricos na Bifurcação do Tronco da Coronária Esquerda: Precisão Matemática versus Complexidade Biológica

**DOI:** 10.36660/abc.20260239

**Published:** 2026-07-22

**Authors:** Guilherme O. Araújo, Henrique Barbosa Ribeiro

**Affiliations:** 1 Hospital das Clínicas Faculdade de Medicina Universidade de São Paulo São Paulo SP Brasil Instituto do Coração do Hospital das Clínicas da Faculdade de Medicina da Universidade de São Paulo, São Paulo, SP – Brasil

**Keywords:** Angiografia Coronária, Ultrassonografia de Intervenção, Angioplastia Coronária com Balão

A geometria das bifurcações coronárias desempenha papel fundamental na fisiopatologia da aterosclerose e no planejamento da intervenção coronária percutânea. Entre essas estruturas, a bifurcação do tronco da coronária esquerda (TCE) assume relevância singular, não apenas pelo amplo território miocárdico irrigado, mas também pela complexa interação entre as forças hemodinâmicas, o remodelamento vascular e a progressão da doença aterosclerótica. Nesse contexto, modelos geométricos que estimam o diâmetro do vaso principal a partir das dimensões dos ramos distais foram propostos como ferramentas auxiliares para o dimensionamento vascular e para a otimização das estratégias intervencionistas. Contudo, faltam estudos comparando, de forma sistemática, esses modelos com o auxílio de ferramentas de imagem intravascular, que são mais precisas na mensuração das artérias coronárias.^1-3^

Garzon et al.^1^ avaliaram a confiabilidade dos modelos de Murray, Finet e Huo-Kassab na estimativa das dimensões da bifurcação do TCE em artérias com aterosclerose moderada, utilizando angiografia coronária quantitativa (QCA) e ultrassom intracoronário (IVUS) como métodos de referência.^1^ Os autores demonstram que os modelos de Murray e Huo-Kassab apresentam maior acurácia e precisão em comparação ao modelo de Finet, com desempenho particularmente superior do modelo de Murray quando o IVUS foi utilizado como padrão de referência. Esses achados estão em consonância com a robustez teórica dos modelos baseados em princípios de dinâmica dos fluidos e otimização energética, em contraste com o modelo de Finet, derivado empiricamente da geometria fractal angiográfica.^1-5^

A superioridade do modelo de Murray observada neste estudo é consistente com evidências experimentais e computacionais que sugerem que a relação cúbica proposta por Murray, mais simples do ponto de vista matemático, pode ser menos sensível a variações nos diâmetros dos ramos distais.^2,4^ Por outro lado, embora o modelo de Huo-Kassab incorpore princípios fisiológicos semelhantes, sua formulação matemática mais complexa pode amplificar pequenas imprecisões nas medidas dos ramos distais, particularmente em artérias com aterosclerose, nas quais o remodelamento vascular altera a geometria original. Esse achado suscita hipóteses relevantes acerca da robustez de modelos matematicamente mais simples em cenários clínicos caracterizados por variabilidade anatômica e pela presença de doença aterosclerótica.

Outro aspecto relevante do estudo é a diferença sistemática entre as medidas obtidas por IVUS e QCA, com o IVUS tendendo a fornecer diâmetros maiores. Essas diferenças refletem as limitações conhecidas da angiografia bidimensional na avaliação do lúmen coronário e são concordantes com estudos prévios que demonstram maior acurácia da imagem intracoronária na caracterização do diâmetro vascular e da carga de placa.^6-10^ Nesse sentido, o IVUS permanece como padrão de referência para o dimensionamento do TCE, estando associado a melhores desfechos clínicos quando utilizado para guiar intervenções, em especial nos procedimentos de maior complexidade, incluindo o TCE.^6-8^

Apesar do desempenho relativamente satisfatório, nenhum dos modelos reproduziu com exatidão as dimensões observadas, com diferenças que podem atingir magnitudes clinicamente relevantes. Em intervenções no TCE, discrepâncias aparentemente pequenas podem resultar em subexpansão ou superdimensionamento dos stents, aumentando o risco de malaposição, dissecção e reestenose, eventos associados a piores desfechos clínicos.^5-7^ Nesse sentido, embora matematicamente elegantes, os modelos geométricos devem ser interpretados como ferramentas auxiliares, e não como substitutos da avaliação direta por imagem intracoronária.

Estudos prévios demonstram que nenhuma lei de escala é capaz de equilibrar completamente as forças hemodinâmicas em bifurcações coronárias reais, o que ressalta que tais modelos representam aproximações da complexa interação entre anatomia, fluxo e doença aterosclerótica.^4,11^ Ainda assim, em ambientes com acesso limitado à imagem intracoronária, essas ferramentas podem contribuir para a identificação de desvios geométricos e fornecer estimativas iniciais para o dimensionamento vascular. Esse aspecto torna ainda mais relevante o presente estudo em países em desenvolvimento como o Brasil, com acesso limitado a ferramentas de imagem intravascular.

À medida que a cardiologia intervencionista evolui em direção à personalização terapêutica, a integração entre modelagem computacional, imagem intracoronária e avaliação fisiológica configura-se como um caminho promissor para o planejamento de intervenções em bifurcações complexas. Estudos futuros com amostras maiores, reconstruções tridimensionais e incorporação de variáveis relacionadas à carga aterosclerótica poderão esclarecer se modelos ajustados à doença arterial podem proporcionar maior precisão e relevância clínica.

Apesar das limitações inerentes ao estudo de Garzon et al.^1^, incluindo o tamanho amostral relativamente reduzido, a análise restrita a bifurcações do TCE com doença arterial coronária de grau moderado e a ausência de segmentos livres de doença ou com carga aterosclerótica extensa — o trabalho acrescenta contribuições relevantes à literatura. Os resultados demonstram que, em bifurcações do TCE com aterosclerose moderada, os modelos geométricos de Murray e de Huo-Kassab apresentam melhor desempenho em relação ao modelo de Finet, com destaque para o modelo de Murray, quando comparados às medidas obtidas por IVUS.

Todavia, entre a elegância dos modelos matemáticos e a complexidade da biologia vascular, permanece clara a necessidade de integrar tais constructos teóricos às modalidades contemporâneas de imagem intravascular, de modo a aprimorar a precisão do planejamento e a segurança das intervenções coronárias. Nesse contexto, o estudo também reforça o papel central da imagem intravascular na prática clínica, em consonância com as recentes atualizações das diretrizes internacionais, que recomendam sua utilização rotineira na otimização dos procedimentos coronários complexos.
