# Supplementary material for: Modelos Geométricos na Bifurcação do Tronco da Coronária Esquerda: Precisão Matemática versus Complexidade Biológica
Source: Arq Bras Cardiol. 2026 Jul 22;123(7):e20260239. [Article in Portuguese] doi: 10.36660/abc.20260239 (PMC13399164; doi:10.36660/abc.20260239)
Supplement: Translation

# Translation: Geometric Models in Left Main Coronary Bifurcation: Mathematical Accuracy and Clinical Limits in the Era of Intravascular Imaging

**DOI:** 10.36660/abc.20260239i

**Authors:** Guilherme O. Araújo, Henrique Barbosa Ribeiro

**Affiliations:** 1 Brazil Instituto do Coração do Hospital das Clínicas da Faculdade de Medicina da Universidade de São Paulo, São Paulo, SP – Brazil

**Keywords:** Coronary Angiography, Interventional Ultrasonography, Angioplasty, Balloon, Coronary

The geometry of coronary bifurcations plays a fundamental role in the pathophysiology of atherosclerosis and in the planning of percutaneous coronary intervention. Among these structures, the bifurcation of the left main coronary artery (LMCA) assumes singular relevance, not only due to the large myocardial territory it irrigates, but also due to the complex interaction between hemodynamic forces, vascular remodeling, and the progression of atherosclerotic disease. In this context, geometric models that estimate the diameter of the main vessel from the dimensions of the distal branches have been proposed as auxiliary tools for vessel sizing and for optimizing interventional strategies. However, studies systematically comparing these models with intravascular imaging tools, which are more precise in measuring coronary arteries, are lacking.^1-3^

Garzon et al.^1^ evaluated the reliability of the Murray, Finet, and Huo-Kassab models in estimating the dimensions of the LMCA bifurcation in arteries with moderate atherosclerosis, using quantitative coronary angiography (QCA) and Intravascular ultrasound (IVUS) as reference methods.^1^The authors demonstrated that the Murray and Huo-Kassab models exhibit greater accuracy and precision compared to the Finet model, with the Murray model performing particularly better when IVUS is used as the reference standard. These findings are consistent with the theoretical robustness of models based on fluid dynamics principles and energy optimization, in contrast to the Finet model, which is empirically derived from angiographic fractal geometry.^1-5^

The superiority of Murray’s model observed in this study is consistent with experimental and computational evidence suggesting that the cubic relationship proposed by Murray, simpler from a mathematical point of view, may be less sensitive to variations in the diameters of the distal branches.^2,4^On the other hand, although the Huo-Kassab model incorporates similar physiological principles, its more complex mathematical formulation may amplify small inaccuracies in the measurements of distal branches, particularly in arteries with atherosclerosis, where vascular remodeling alters the original geometry. This finding raises relevant hypotheses about the robustness of mathematically simpler models in clinical scenarios characterized by anatomical variability and the presence of atherosclerotic disease.

Another relevant aspect of the study is the systematic difference between the measurements obtained by IVUS and QCA, with IVUS tending to provide larger diameters. These differences reflect the known limitations of two-dimensional angiography in evaluating the coronary lumen and are consistent with previous studies that demonstrate greater accuracy of intravascular imaging in characterizing vascular diameter and plaque burden.^6-10^In this sense, IVUS remains the gold standard for assessing LMCA, being associated with better clinical outcomes when used to guide interventions, especially in more complex procedures, including LMCA.^6-8^

Despite relatively satisfactory performance, none of the models accurately reproduced the observed dimensions, with differences that can reach clinically relevant magnitudes. In LMCA interventions, seemingly small discrepancies can result in underexpansion or oversizing of the stents, increasing the risk of malposition, dissection, and restenosis, events associated with worse clinical outcomes.^5-7^In this sense, although mathematically elegant, geometric models should be interpreted as auxiliary tools, and not as substitutes for direct evaluation by intravascular imaging.

Previous studies demonstrate that no scaling law is capable of completely balancing hemodynamic forces in real coronary bifurcations, highlighting that such models represent approximations of the complex interaction between anatomy, flow, and atherosclerotic disease.^4,11^Nevertheless, in settings with limited access to intravascular imaging, these tools can contribute to the identification of geometric deviations and provide initial estimates for vascular sizing. This aspect makes the present study even more relevant in developing countries like Brazil, with limited access to intravascular imaging tools.

As interventional cardiology evolves toward therapeutic personalization, the integration of computational modeling, intravascular imaging, and physiological assessment emerges as a promising path for planning interventions at complex bifurcations. Future studies with larger samples, three-dimensional reconstructions, and the incorporation of variables related to atherosclerotic burden may clarify whether models adjusted for arterial disease can provide greater accuracy and clinical relevance.

Despite the inherent limitations of the study by Garzon et al.^1^, including the relatively small sample size, the analysis restricted to LMCA bifurcations with moderate coronary artery disease, and the absence of disease-free segments or segments with extensive atherosclerotic burden — this work makes relevant contributions to the literature. The results demonstrate that, in LMCA bifurcations with moderate atherosclerosis, the Murray and Huo-Kassab geometric models perform better than the Finet model, particularly the Murray model, when compared to measurements obtained by IVUS.

However, between the elegance of mathematical models and the complexity of vascular biology, the need to integrate such theoretical constructs with contemporary intravascular imaging modalities remains clear, in order to improve the precision of planning and the safety of coronary interventions. In this context, the study also reinforces the central role of intravascular imaging in clinical practice, in line with recent updates to international guidelines, which recommend its routine use in optimizing complex coronary procedures.
